# Recent Advances in the Diagnosis and Management of Pediatric Orthopedic Disorders: A Comprehensive Review

**DOI:** 10.7759/cureus.95580

**Published:** 2025-10-28

**Authors:** Jitender Saini, Nischay Kaushik, Prerak Jain, Juvesh Kumar, Bhanupriya Singh

**Affiliations:** 1 Department of Orthopaedics, Dr. Baba Saheb Amedkar Medical College and Hospital, New Delhi, IND; 2 Department of Radiodiagnosis, Sanjay Gandhi Postgraduate Institute of Medical Sciences, Lucknow, IND

**Keywords:** artificial intelligence, biologic augmentation, growth preservation, pediatric orthopedics, regenerative medicine

## Abstract

Pediatric orthopedic disorders, spanning congenital, developmental, traumatic, infectious, and neoplastic conditions, are a major source of preventable disability worldwide. Despite notable advances over the past decade, progress in diagnostic precision, surgical innovation, and regenerative therapy development often remains compartmentalized within subspecialties, limiting the translation of innovations across the field. This review aims to summarize recent advances in pediatric orthopedic diagnosis and treatment, assess emerging technologies such as AI-assisted imaging and regenerative medicine, and highlight key challenges and evidence gaps to guide future practice. A structured literature search was conducted across major biomedical databases (PubMed, Scopus, and Web of Science) to identify studies focused on pediatric populations, emphasizing multicenter cohorts and randomized controlled trials (RCTs) when available. Key advances include radiation-sparing diagnostic tools such as artificial intelligence (AI)-assisted ultrasonography, low-dose cone-beam computed tomography (CBCT), and positron emission tomography/magnetic resonance imaging (PET/MRI); minimally invasive, growth-preserving interventions like guided growth, bioabsorbable fixation, and patient-specific three-dimensional (3D)-printed implants; and biologic augmentation approaches using platelet-rich plasma (PRP), mesenchymal stem cells (MSCs), and scaffold-based regeneration. While these innovations enhance diagnostic precision, reduce morbidity, and preserve skeletal potential, most are underpinned by Level II/III evidence and face barriers related to cost, access, and resource availability. By integrating cross-disciplinary progress and identifying transferable strategies, this review offers clinicians, researchers, and policymakers an actionable roadmap to align technological innovation with robust evidence generation and equitable delivery, ensuring sustainable improvements in musculoskeletal health for children globally.

## Introduction and background

Pediatric orthopedic conditions in children are some of the most treatable yet common causes of preventable disability globally, affecting millions each year and leading to significant long-term ramifications on musculoskeletal health [1]. These disorders cut across congenital malformations like clubfoot and discrepancies in limb length, developmental malformations like developmental dysplasia of the hip (DDH), and acquired conditions like fractures, sports injuries, infections, and bone tumors [2]. Congenital clubfoot is estimated to occur globally at a rate of 1 to 2 per 1,000 live births, and pediatric fractures can make up to 30% of all fractures in growing populations, with 15 to 20% involving the growth plate [3]. With the increase in organized sports, overuse and acute injury in youth athletes are on the rise, highlighting the changing epidemiological pattern of orthopedic care in pediatrics [4]. This review provides a comprehensive synthesis of recent diagnostic and therapeutic advancements in pediatric orthopedics, evaluating emerging technologies and highlighting persistent gaps that influence clinical outcomes and research priorities.

The diagnostic methods in the field have experienced a phenomenal change [5]. Although conventional radiography remains a first-line tool, imaging technology has advanced to the level of earlier and more specific detection [6]. The use of high-resolution ultrasonography has emerged as the cornerstone of early screening of DDH, with sensitivities of 90% or greater in population programs [7]. Magnetic resonance imaging (MRI), especially using cartilage-sensitive sequences, can be used to evaluate physeal injuries, soft-tissue pathology, and inflammatory changes in detail in a non-radiation-exposed setting, which is critical in patients at pediatric ages [8]. The use of low-dose CT protocols is still important in the mapping of complex fractures; however, they should be used selectively [9]. Artificial intelligence (AI)-assisted platforms, which have been validated in recent multicenter trials, have shown diagnostic accuracies of over 92% in pediatric fracture identification, automated scoliosis angle measurements, and growth plate analysis [10]. Molecular diagnostic technologies such as polymerase chain reaction (PCR) panels and next-generation sequencing are facilitating the rapid distinction between bacterial and viral etiology in osteoarticular infections, which has enhanced the accuracy of the diagnosis and the time of treatment [11].

**Figure 1:**
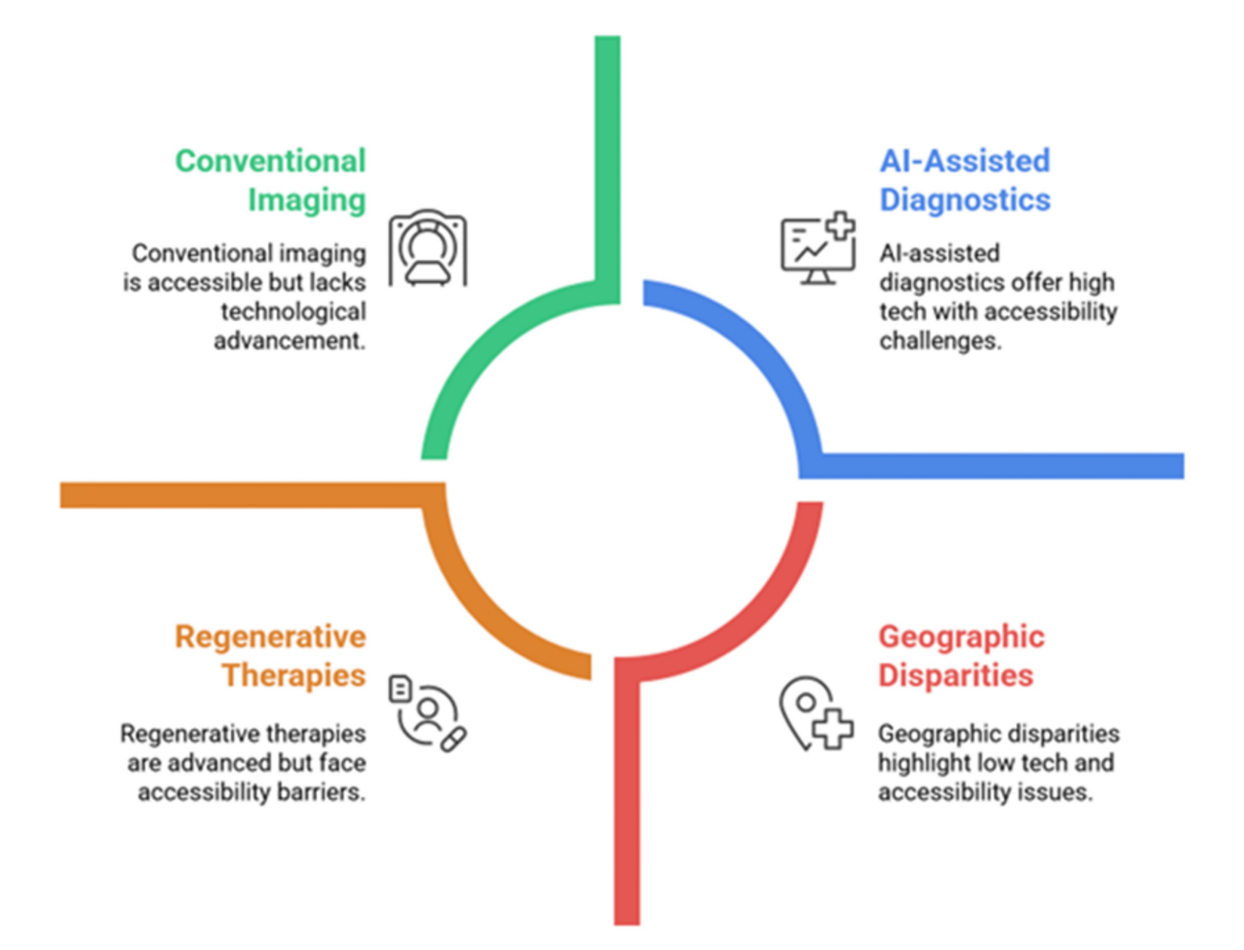
Key technological and systemic trends in pediatric orthopedic care The image is created by the authors. Figure 1 illustrates four major domains influencing current pediatric orthopedic care. (1) Conventional imaging remains reliable and widely used but offers limited technological advancement. (2) AI-assisted diagnostics represent high-tech innovations that enhance diagnostic precision yet face challenges related to reliability, data validation, cost, and accessibility. (3) Geographic disparities highlight unequal access to advanced diagnostic and therapeutic tools between high- and low-resource settings. (4) Regenerative therapies demonstrate rapid technological progress but are constrained by regulatory barriers, high costs, and limited pediatric-specific clinical evidence.

The management of developmental dysplasia of the hip (DDH) and other pediatric orthopedic conditions has changed to emphasize precision, preservation of biology, and minimally invasive intervention [12]. Tension band plate-based guided growth methods are able to correct angular deformities gradually, without osteotomy, and have shorter recovery times and complication rates [13]. Robotically assisted navigation is also enhancing screw position accuracy during complex spinal deformity surgery, and arthroscopic techniques are reducing morbidity during joint preservation surgery [14]. Fracture fixation with bioabsorbable implants has demonstrated similar stability to metallic hardware with the added benefit of avoiding surgery to remove implants, a major benefit in children [15]. Mesenchymal stem cell implantation and platelet-rich plasma therapy are regenerative solutions in the early stages of clinical trials and show promise in cartilage regeneration and faster fracture healing [16]. Multidisciplinary rehabilitation strategies with gait analysis, the use of physiotherapy to treat affected parts, and psychosocial support have been shown to enhance postoperative outcomes, particularly in children with neuromuscular disorders such as cerebral palsy. Figure 1 shows the overlap of the developments in imaging, diagnostics, and regenerative therapies with geographic disparities in access, outcomes, and equity of pediatric orthopedic care.

Despite these innovations, access to advanced diagnostic technologies, minimally invasive surgical options, and regenerative therapies remain uneven. Although the use of AI-assisted MRI interpretation is possible in high-income countries, including Japan, Germany, and the United States, this technology is not accessible in more than 70% of low- and middle-income areas, where plain radiography is still the primary means of diagnosis [17]. Promising as they may be, regenerative therapies remain largely experimental in pediatrics and are limited by regulatory, ethical, and cost limitations. The evidence base is also limited: randomized controlled trials in children are few because of the recruitment difficulties, ethical issues, and the need to follow up in the long term. As a result, the level of practice variation is still high, as there are regional differences in the screening of DDH, timing of surgery in scoliosis, and use of antibiotics in osteoarticular infection.

The motivation behind this review is the duality of the current state of rapid technology development and uneven clinical translation. The literature is well-developed in terms of focus on individual disorders or individual innovations, but integrative syntheses that relate the most recent diagnostic modalities with the current management strategies along the full range of pediatric orthopedic conditions are lacking. While adult orthopedic literature is abundant, pediatric evidence often lags behind, forcing clinicians to extrapolate adult data, a risky approach that overlooks key developmental, anatomical, and physiological differences in children.

Objectives for the review

This review aims to synthesize recent diagnostic and therapeutic advances across major pediatric orthopedic conditions, evaluate emerging technologies such as AI-assisted imaging, robotic surgery, and regenerative medicine for their current and potential clinical roles, and identify key challenges, evidence gaps, and research priorities to guide future practice. It integrates these innovations into a unified, evidence-based framework that highlights both the opportunities and the persistent barriers in modern pediatric orthopedics.

Methodological considerations

This article is a narrative review that used a structured literature search to capture recent advances in pediatric orthopedic disorders. Searches were performed in PubMed, Scopus, and Web of Science for the years 2015 through 2025, using combinations of relevant keywords and Medical Subject Headings (MeSH) terms, which are standardized indexing terms used by PubMed to improve search precision and ensure inclusion of all related concepts.

The search terms included “pediatric orthopedic disorders,” “congenital limb deformities,” “developmental dysplasia of the hip,” “pediatric fractures,” “scoliosis,” “osteomyelitis,” “septic arthritis,” “neuromuscular disorders,” “cerebral palsy,” “bone tumors,” “regenerative medicine,” “stem cells,” “robotic surgery,” and “artificial intelligence.” These terms were selected to encompass the full spectrum of pediatric orthopedic practice, from congenital and developmental abnormalities to traumatic, infectious, neoplastic, and technology-driven innovations. Reference lists of key articles were also screened to identify additional studies.

A total of 51 studies met the inclusion criteria, representing randomized controlled trials (RCTs), multicenter cohort studies, and high-quality observational research. Greater weight was assigned to RCTs and multicenter data when summarizing evidence, while observational and narrative sources were used to provide context or highlight emerging trends.

Eligible publications were peer-reviewed, written in English, involved human participants under 18 years of age, and included original research, high-quality reviews, or observational studies. Animal studies, adult-only data, conference abstracts, and case reports except for rare conditions or novel treatment approaches were excluded. This review is narrative in nature; therefore, a PRISMA flow diagram and formal risk-of-bias tables are not provided. Evidence from the included studies was summarized descriptively to highlight diagnostic modalities, management strategies, outcomes, and limitations.

## Review

Congenital limb deformities

Birth defects of the limbs are a continuum of malformations that range throughout the spectrum from mild angular deformities to gross structural inadequacies [18]. These conditions can be isolated and found in complex syndromes and may involve upper or lower limbs [19]. The prevalence in the world is broadly varied, with common deformities like clubfoot having an incidence of about 1-2 per 1000 live births and rare deficiencies like fibular hemimelia having an incidence of 7.4 per million [20]. The etiology is multifactorial and includes genetic factors, intrauterine mechanical limitation, vascular interference during limb bud formation, and teratogenic exposure. Early identification enables the best timing of remedial action to utilize the remaining developmental potential of the child to reduce long-term disability [21]. Prenatal ultrasonography remains the cornerstone of antenatal detection. Long-bone length discrepancies, joint malalignment, or missing skeletal components may be detected on standardized fetal anomaly scans, which are usually done between 18 and 22 weeks of gestation [22]. Major long-bone anomalies are detected in more than 85% of experienced centers [23]. The development of the three-dimensional (3D) and four-dimensional (4D) ultrasound technology gives a more realistic image of the spatial orientation and fetal movement. Real-time observation of the motion of the joints allows distinguishing between fixed deformity and positional limitations and informs prognostication and parental discussion [18].

Postnatal imaging is based on antenatal results, especially in neonates whose bones are not fully ossified. Fast spin-echo and gradient-echo sequences of MRI provide a precise visualization of cartilaginous structures, integrity of physis, and periarticular soft tissues without the use of ionizing radiation [15]. This is particularly useful in conditions such as congenital knee dislocation, in which the quadriceps contracture and joint incongruity determine surgical planning. Whole-body MRI has been a valuable addition to the syndromic presentation to identify subtle related anomalies so that both limb and systemic issues are addressed in the management [24]. Correction strategies in surgery have gone to modulating the growth and preserving the tissue. Guided growth is achieved through temporary hemiepiphysiodesis, in which tension-band plates or staples are used to gradually correct angular deformities by selectively limiting one side of the growth plate. Average correction rates are 0.5-1° per month, and results depend on patient age, residual growth, and severity of deformity [25]. This less invasive procedure is reversible, which minimizes the necessity of huge osteotomies in suitable situations.

**Figure 2:**
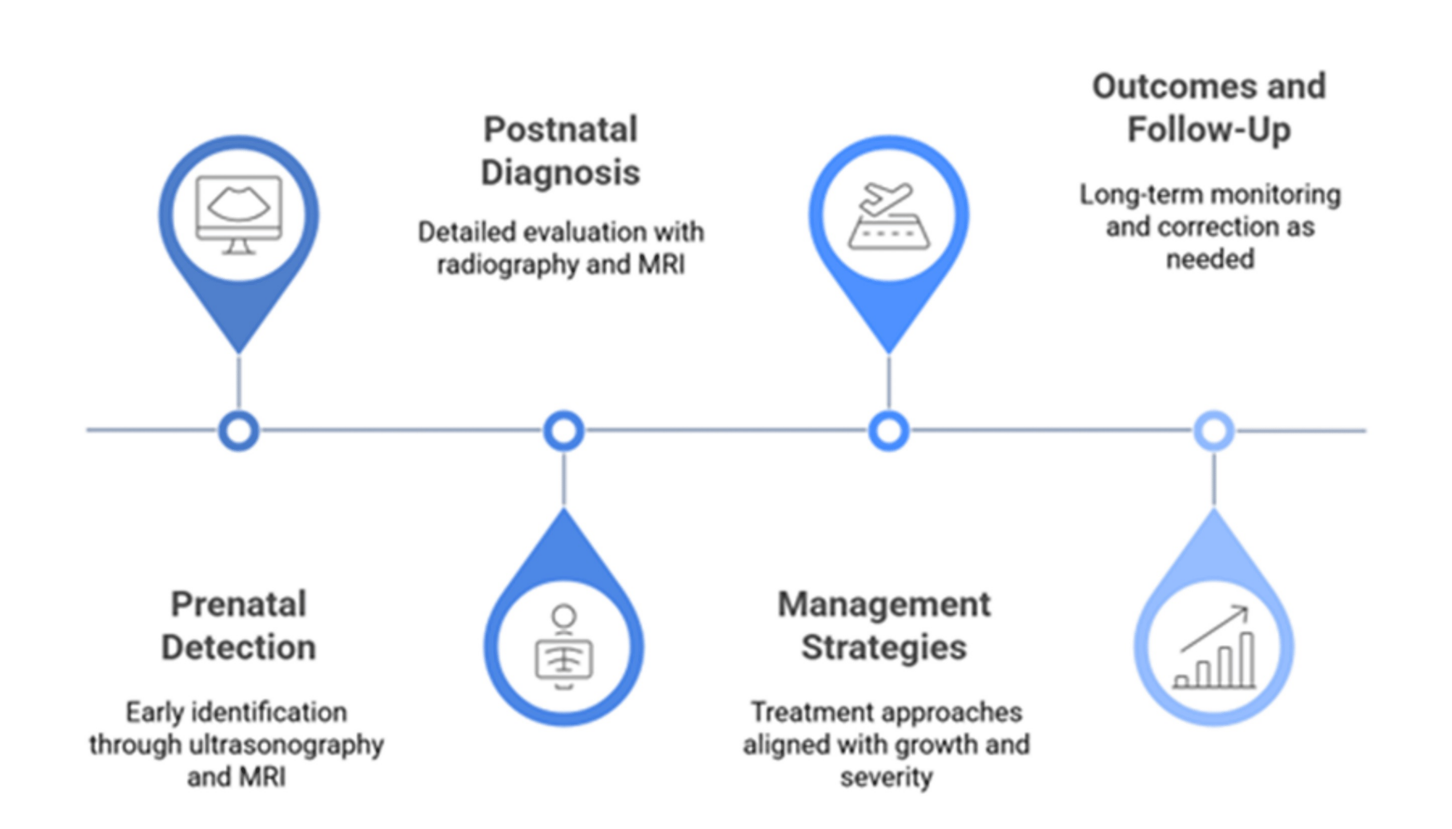
Sequential pathway for diagnosis and management of congenital limb deformities The image is created by authors.

In situations of complex deformities with angular correction and lengthening, distraction osteogenesis is considered the gold standard. External fixators, like the Ilizarov ring system, allow correction gradually in more than one plane, whereas monolateral fixators are linked with higher patient comfort [26]. Motorized intramedullary lengthening nails, including the PRECICE (NuVasive Specialized Orthopedics, Aliso Viejo, California) system, have also been introduced as an alternative to these in older children and adolescents with adequate canal diameter. Such devices can be internally lengthened under control with an external magnetic controller at a rate of 1 mm/day, with less risk of infection and increased patient mobility during treatment [27]. The interaction of epidemiology, diagnostic pathways, and management strategies in congenital limb deformities is shown in Figure 2.

Developmental dysplasia of the hip (DDH)

Another common pediatric orthopedic condition is DDH, which involves acetabular dysplasia to frank dislocation and is known as developmental dysplasia of the hip. The incidence varies geographically, with higher rates reported in certain populations and regions compared to others [28]. Breech presentation, female sex, being the first offspring, oligohydramnios, and positive family history are risk factors. It is important to diagnose in time; otherwise, it may cause chronic pain, gait disturbance, and early-onset osteoarthritis. Screening strategies remain debated [29]. Universal screening with ultrasound, as in Austria and Germany, covers all newborns by standardized procedures and is usually performed at between 4 and 6 weeks of age, using the Graf classification. Such programs have virtually eradicated late-presenting DDH with a current incidence of <0.5 per 1,000 live births [30]. Nevertheless, the strategy requires significant resources and may cause overtreatment because as many as 60% of mild cases of instability spontaneously resolve in a few weeks [7]. Selective screening, used most commonly in North America and the UK, focuses on infants who have risk factors or clinical instability. Although more resource-efficient, it has a low-level risk of a false negative, failing to detect clinically silent pathologic hips, which is documented as 3-5% of cases in some series [31]. New AI-based algorithms that combine both perinatal and demographic information have shown >90% sensitivity in pilot studies and can decrease unnecessary imaging by up to 25% but have yet to be evaluated in large and diverse populations [10].

Management depends on age and severity. The Pavlik harness is still the gold standard in infants of less than six months with reducible hips, with 85-95% success rates in stable dysplasia [32]. The harness keeps the hips in flexion and abduction to encourage acetabular remodeling and permit active movement. More recent dynamic abduction orthoses (such as the Tubingen brace) offer the same therapeutic positioning but with more mobility, making them more comfortable and easier to wear [1]. In randomized comparisons, they indicate comparable success rates with a shorter average treatment duration than the Pavlik harness [33]. Surgical intervention is required when bracing fails or when the diagnosis is late. A less invasive surgery compared to the open approaches has been the arthroscopic-assisted closed reduction. With direct visualization, intra-articular obstructions, including hypertrophied ligamentum teres, pulvinar tissue, or inverted labrum, can be dealt with before reduction, obviating the necessity of widespread open dissection. In early series, stability rates are similar to those of open reduction, and postoperative stiffness rates are lower than one-half [34].

Slipped capital femoral epiphysis (SCFE)

The most prevalent hip condition in the adolescent age group is slipped capital femoral epiphysis, which has an incidence rate of 2-10/100,000, which is higher in boys and those who are obese and have endocrine disorders [35]. The disorder is typified by posterior inferolateral slippage of the femoral epiphysis through the hypertrophic region of the physis, which results in abnormal biomechanics and possible degenerative changes in the joint in the long run in the absence of treatment [36]. Radiographic diagnosis is based on the radiographic appearance of the Klein line and the Southwick angle; however, early or pre-slip stages may not be evident on plain films. MRI is particularly useful in the early identification of alterations like physeal widening, marrow edema, and peri-physeal signal changes [37]. In high-risk patients with uncharacteristic knee or thigh pain, prophylactic fixation can be performed based on MRI detection, decreasing the development of unstable slips from 68 to less than 5% [38]. Such early treatment can avert the gross deformity and impingement of late-stage SCFE.

Stable slips are generally treated with in situ pinning, where a single cannulated screw is inserted perpendicular to the physis to avoid further displacement and to maintain some growth potential [14]. Fully threaded screws offer an improved purchase, and bioabsorbable implants eliminate the necessity of a future hardware removal procedure. Unstable or severe slips may need to be corrected by realignment to recreate the femoral head-neck offset [21]. The modified Dunn surgery is done through surgical dislocation, and it enables anatomic reduction in direct view without sacrificing the blood supply to the femoral head. Laser Doppler Flowmetry has also shown intraoperative perfusion monitoring capable of decreasing the avascular necrosis rates from 15-20% to less than 7% in specialized centers [39].

Pediatric fractures

Musculoskeletal injuries are the most common injuries in children, and fractures constitute 25-30% of all pediatric trauma in large epidemiologic studies [40]. The immature skeleton has a biological and biomechanical environment with open physes, a thicker and more metabolically active periosteum, and increased remodeling potential that leads to a different pattern of fractures and a different healing response compared to adults [19]. Correct classification is important in order to predict growth disturbances and prevent long-term deformities. Diagnostic imaging has improved significantly over the past decade, and there has been a transition to imaging that is as detailed as possible with as little radiation as possible [26]. The volumetric reconstructions of low-dose three-dimensional imaging, especially cone-beam computed tomography (CBCT), currently offer radiation savings of up to 78% of conventional CT, as seen in a multicentric pediatric trauma study with 312 cases [41]. The technique renders a clear visualization of articular congruity, fracture line morphology, and physeal involvement in such injuries as intra-articular fractures of the distal humerus or transitional ankle fractures [33]. Iterative reconstruction algorithms can allow high-image quality at reduced dose and thus safer serial imaging of complex injuries, a principle that can be applied to scoliosis follow-up and tracking of infections [42].

**Figure 3:**
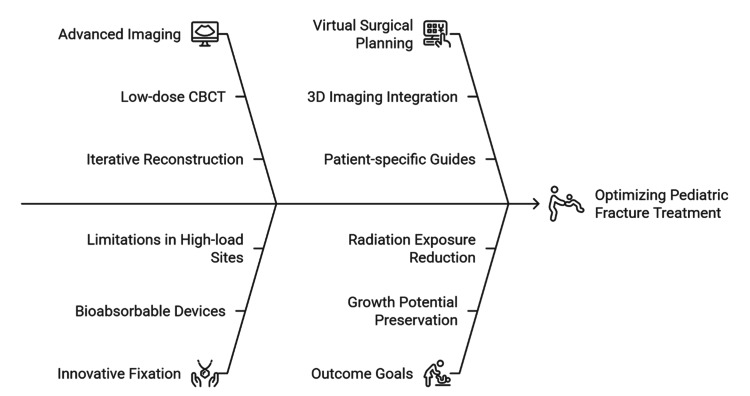
Integrated approach to pediatric fracture management The image is created by authors

The management has put more focus on approaches that will maintain growth potential without repeat surgery. Bioabsorbable constructs, usually made of polylactic acid (PLA) or polyglycolic acid (PGA) composites, offer an initial stability equivalent to stainless steel implants in metaphyseal fractures based on biomechanical testing and mid-term follow-up of cohorts of 100-150 children [43]. Predictably, they are hydrolyzed into glycolic and lactic acid monomers within a period of 6 to 24 months, which are then finally metabolized by the Krebs cycle [4]. Although they do not require the removal of hardware, their effect in high-weight-bearing sites (e.g., femoral shaft) is a point of contention, with temporary inflammatory responses to degradation reported [24]. Low-dose 3D imaging has been combined with computer-assisted modeling to perform virtual surgical planning (VSP) to simulate reduction, optimize fixation strategy, and manufacture patient-specific drill guides. Future controlled studies have demonstrated that VSP may decrease intraoperative fluoroscopy exposure by 35-40% and increase screw trajectory accuracy in complex periarticular fractures [44]. The focus on anatomical mapping before intervention is a common theme throughout the field of pediatric orthopedics, and it connects the treatment of fractures to the contemporary approach of treating scoliosis and treating infections [10]. Figure 3 shows the convergence of advanced imaging, novel fixation, and virtual surgical planning in optimizing the treatment of pediatric fractures.

Scoliosis and spinal deformities

Pediatric spinal deformities, including adolescent idiopathic scoliosis, congenital scoliosis, and early-onset kyphotic curves, present unique challenges, balancing curve correction with preservation of spinal growth and thoracic development [4]. Longitudinal monitoring historically relied on serial radiographs, raising concerns about cumulative radiation exposure throughout childhood and adolescence [15]. Three-dimensional surface topography (3DST) has emerged as a radiation-free alternative [9]. This technology projects structured light or laser patterns onto the torso to create a 3D surface model, from which parameters like trunk rotation and rib hump prominence are derived [18]. Correlation studies involving more than 400 children (2018-2023) have demonstrated strong agreement between surface indices and Cobb angles, with sensitivities above 85% for detecting ≥5° progression [6]. The absence of radiation enables more frequent assessments and early detection of brace failure, akin to the role of low-dose imaging in fracture surveillance [11].

In surgical management, growth-friendly instrumentation has been transformed by magnetically controlled growing rods (MCGRs) [2]. Each MCGR contains an internal magnet linked to a lead screw, which lengthens when an external magnetic controller induces rotation [14]. This design permits noninvasive outpatient distractions, avoiding the 6-9 monthly open lengthenings required by traditional rods [8]. Multicenter registry data from 547 patients (mean follow-up 4.2 years) show that MCGRs achieve mean Cobb angle reductions of 44-47%, equivalent to traditional systems, while reducing unplanned surgical procedures by 73% [19]. Patient-reported outcomes also suggest improved comfort and reduced school absence [7]. However, MCGRs are not without limitations [1]. Mechanical failure rates range from 10-15%, most commonly actuator wear or rod fracture, and distraction gains may plateau in stiff curves [13]. Long-term risks include junctional kyphosis and curve progression after growth completion, emphasizing the need for structured post-MCGR follow-up [3]. Comparative analyses indicate that while both MCGR and traditional growing rods achieve similar final curve correction, MCGRs offer superior quality-of-life metrics and lower cumulative morbidity [10]. The trade-off is high initial device cost, a barrier especially in low-resource health systems, paralleling access limitations seen with advanced fracture fixation and molecular diagnostics in infection care [17].

Pediatric osteomyelitis and septic arthritis

Acute hematogenous osteomyelitis and septic arthritis are acute infections in children that, unless treated promptly, may result in permanent structural damage and permanent disability [18]. Their incidence is not uniform around the world, but the effects of delayed treatment are always devastating, especially in young children with open growth plates [12]. The early symptoms in infants are usually subtle, and the routine inflammatory markers are not very specific, which complicates clinical recognition [21]. The current developments have changed the degree of certainty of diagnosis as well as the effectiveness of therapeutic interventions [14]. The integration of molecular assays has significantly reduced the time it takes to identify the pathogen, which allows the administration of customized antimicrobial treatment in situations where previous antibiotics would have caused cultures to become negative [23]. The concurring development of therapy approaches to shorter intravenous regimens with early switch to oral drugs has minimized hospitalization without sacrificing cure rates, with strong evidence in children [16].

It is also crucial to realize that the best results go beyond eliminating infections [19]. Structured follow-up and early physiotherapy-guided mobilization have now become part of management pathways to ensure joint functionality is maintained, avoid stiffness, and monitor sequelae related to growth [25]. This progression is a prime example of a paradigm shift in the management of pediatric musculoskeletal infections to include rapid diagnostics, evidence-based antibiotic stewardship, and proactive rehabilitation in a comprehensive model that treats the patient as a whole [13]. Table 1 provides the existing evidence-based strategies that enhance diagnosis, treatment, and outcomes in pediatric osteomyelitis and septic arthritis [20].

**Table 1:** Diagnostic and therapeutic advances in pediatric osteomyelitis and septic arthritis The table was created by the authors using data summarized from the literature cited within the table ([37,23,29,17,31,5]). It is an original synthesis and is not a verbatim reproduction of any published table; no publisher permissions are required.

Domain	Key Advances / Evidence	Impact	Clinical Implications	References
Epidemiology	Incidence: 1–13 per 100,000 children, varies by geography	Establishes disease burden and urgency for timely intervention	Raises awareness for early recognition, particularly in high-incidence regions	[37]
Diagnostic Challenges	Early signs are subtle in infants; CRP and ESR are elevated but nonspecific.	Risk of delayed diagnosis leading to cartilage damage, growth arrest, and chronic disability	Necessitates high clinical suspicion and integration with advanced diagnostics	[23]
Molecular Diagnostics	Multiplex PCR: Detects bacterial DNA from synovial fluid/bone aspirate within hours; sensitivity >90% in culture-negative cases (prospective studies, n >200) NGS: Identifies polymicrobial and difficult-to-culture organisms, effective even after partial antibiotic therapy	Rapid pathogen identification guides targeted therapy; it overcomes culture limitations.	Enables the timely initiation of targeted antibiotics, reducing unnecessary broad-spectrum use	[29]
Therapeutic Regimens	Osteomyelitis: RCTs (2015–2022, n=80–120) show early oral switch after 3–7 days IV is as effective as 4–6 weeks IV (<5% relapse) Septic Arthritis: 10–14 days total antibiotics for uncomplicated cases with prompt drainage	Shorter hospital stay; reduced cost and morbidity; maintains cure rates	Supports protocol change toward shorter IV courses and outpatient management in stable patients	[17]
Rehabilitation	Mobilization within 48–72h post-drainage under physiotherapy supervision	Preserves joint motion, muscle strength; does not increase reinfection risk	Encourages early physiotherapy integration into care pathways	[31]
Long-term Outcomes	Full recovery in most uncomplicated cases within 6–12 months; long-term follow-up detects residual stiffness or growth disturbance	Ensures optimal function and early intervention for sequelae	Justifies structured follow-up to identify and treat late complications	[5]

Integrative summary: unifying trends and translational imperatives

In pediatric fractures, scoliosis, and musculoskeletal infections, there are a number of common themes that are applicable across the field of pediatric orthopedics and that characterize the current direction of pediatric orthopedic care [27]. First among these is the importance of early, high-fidelity diagnosis, attained by modalities like low-dose CBCT, radiation-free 3DST, and rapid molecular pathogen assays, that permits timely, targeted responses before the development of irreversible pathology [21]. Within both fields, the most influential innovations integrate technological accuracy with minimally invasive, growth-sparing approaches: bioresorbable implants that give stable fixation and then dissolve without the need to be removed, magnetically controlled growing rods that enable noninvasive lengthening of the spine, and simplified antibiotic treatment, which cures in fewer days in the hospital [29].

One important comparative observation is that these advanced modalities often compare favorably to or even exceed the traditional methods in terms of safety and patient satisfaction but are always limited by cost, special training, and infrastructure demands-factors that can restrict implementation outside of the more resource-endowed facilities [22]. In addition, the available evidence base is encouraging, but most of it is due to observational cohorts and multicenter registries; there are few high-quality and pediatric-specific randomized controlled trials [30]. These advances will not translate to broad clinical benefit merely through technological uptake [24]. It demands advanced patient selection criteria, standard postoperative monitoring protocols, and models that could be used in various healthcare settings [28]. There will be a need to have fair distribution, which will be enabled by the alignment of health policies and investment in training of the workforce [26]. It is not just that the new paradigm in pediatric orthopedics is a matter of technological sophistication but of integrating precision, minimal invasiveness, and equity into a new paradigm of integrated care, an aspiration that can be achieved only when sophistication is accompanied by vigorous, evidence-based implementation plans [20].

Neuromuscular disorders in children: cerebral palsy-related orthopedic issues

Children with neuromuscular diseases, especially cerebral palsy (CP), are the most common cause of functional disability and orthopedic morbidity. CP occurs in about 2-3/1000 live births worldwide and is more common in preterm and low-birthweight infants [16]. Pathophysiology is one of the nonprogressive brain damage that results in abnormal tone of the muscle, poor motor control, and secondary musculoskeletal deformities. Involvement of the lower limbs can lead to crouch gait, hip displacement, equinus contracture, and rotational malalignment.

Gait analysis and motion capture

Current gait labs combine 3D motion capture, force plates, and surface electromyography to deliver a quantitative characterization of gait kinematics, kinetics, and muscle activity [29]. Reflective markers are placed on anatomical points and tracked using infrared cameras to determine the trajectory of the joints throughout a gait cycle [27]. This will enable distinction of primary deformities and compensatory adaptations critical in the surgical planning [25]. A future multicenter study (n = 312 children with CP, GMFCS [Gross Motor Function Classification System] levels II-IV) indicated that including gait analysis in preoperative planning altered surgical planning in 40% of cases and led to higher Gillette Functional Assessment scores at two-year follow-up as compared to preoperative planning based on physical examination alone [30].

Selective dorsal rhizotomy (SDR)

SDR treats the neurogenic aspect of spasticity by selective transection of overactive dorsal sensory rootlets in the lumbosacral spinal cord [36]. This inhibits Ia afferent input to spinal reflex arcs, which decreases muscle tone but does not influence voluntary motor pathways. SDR has been linked to long-term improvements in spasticity, improved gait symmetry, and reduced subsequent orthopedic surgery in ambulatory children in long-term cohort studies with 10-15 years of follow-up (n > 150) [40].

Single-event multi-level orthopedic surgery (SEMLS)

Based on gait analysis, SEMLS remedies several fixed contractures and bony deformities during a single session. Hamstring lengthening, rectus femoris transfer, femoral derotation osteotomy, and foot deformity correction are common surgeries [22]. SEMLS uses fewer anesthesia exposures and rehabilitation cycles than the staged ones. Five-year follow-up (n = 142) demonstrated that SEMLS led to better step length, cadence, and patient-reported function than single-level procedures [9]. Gait labs and multidisciplinary CP surgical programs are concentrated in high-income countries in tertiary centers. In a lot of low-resource environments, observational gait assessment and staged procedures are the standard, which restricts the possibility of achieving optimal timing and integration of interventions.

Bone tumors in children

Uncommon, primary malignant bone tumors in children, most commonly osteosarcoma and Ewing sarcoma, are among the most aggressive malignancies seen in pediatric oncology [15]. Their highest rate of occurrence among adolescents means that they are associated with times of active skeletal development, and therefore, early diagnosis and treatment are important to optimize survival and functional outcomes [29]. The standard is multimodal therapy, where chemotherapy and surgical resection are the backbone of treatment, and where possible limb salvage is being increasingly used instead of amputation in a situation where oncological margins can be maintained [20]. Local staging and treatment planning have been improved significantly with advances in diagnostic imaging [13]. The combination of metabolic and anatomical evaluation during the same PET/MRI session increases the characterization of the lesions, decreases radiation exposure, and lessens the necessity of several anesthetics in younger patients [27]. Modality is especially useful in differentiating viable tumor tissue and changes after treatment, which can be a problem that makes decision-making difficult when only using conventional imaging [16].

Surgical reconstruction has also been advancing, with the introduction of patient-specific 3D-printed endoprostheses [23]. With their superior mechanical reliability and better functional symmetry during long-term follow-up, these implants, which are shaped to fit resection defects perfectly and allow skeletal growth, are proving to be superior to standard modular systems [30]. Nevertheless, these innovations are mainly confined to the well-funded healthcare systems, and cost, infrastructure, and manufacturing barriers restrict their applicability to low-income environments [18]. Covering this gap is an essential issue in providing equitable access to state-of-the-art pediatric oncology care [25]. Table 2 illustrates innovations in diagnostic and reconstructive approaches to pediatric bone tumors as well as resource-poor challenges.

**Table 2:** Diagnostic innovations, surgical reconstruction advances, and resource-related challenges in the management of pediatric bone tumors The table was created by the authors using data summarized from the literature cited within the table ([3,11,35,20]). This table is an original synthesis and is not a verbatim reproduction of any published table; no publisher permissions are required.

Domain	Key Advances/Evidence	Impact	Clinical Implications	References
Epidemiology	Osteosarcoma and Ewing sarcoma: 6–8% of pediatric cancers; peak in adolescence	Rare but aggressive tumors	Require early detection and aggressive multimodal care	[3]
PET/MRI	Combines ^18F-FDG PET with MRI; 94% sensitivity, 91% specificity; 72% less radiation vs PET/CT.	Improves staging accuracy; reduces radiation/anesthesia	Preferred over PET/CT for local staging, where available	[11]
3D-Printed Endoprostheses	Patient-specific titanium prostheses; expandable designs; 30% fewer mechanical failures vs standard	Precise reconstruction; accommodates growth	Suitable for skeletally immature patients in high-resource settings	[35]
Low-Resource Settings	PET/MRI and custom implants are often unavailable; high cost	Reliance on modular prostheses; limited customization	Need for affordable, scalable solutions	[20]

Sports-related injuries in youth athletes

Single-sport specialization has led to overuse and acute injuries year-round in youth athletes, such as anterior cruciate ligament (ACL) tears, osteochondritis dissecans (OCD), and physeal stress injuries. Management involves a balance between tissue healing and protection of growing open growth plates and avoiding early degenerative changes.

Wearable sensor technology

Inertial measurement units (IMUs), wearable IMUs that include accelerometers and gyroscopes, measure accelerations of the limb and angular velocities during sport-specific movements [10]. This data is processed by algorithms to estimate joint loads, asymmetry indices, and high-risk movement patterns. Elbow valgus torque based on IMUs has a high correlation with ulnar collateral ligament strain in youth baseball and allows workload adjustment to avoid injury [24]. A randomized controlled trial of female adolescent soccer players (n = 234) who received wearable load feedback as part of training programs found a 38% decrease in lower-limb non-contact injuries during a competitive season compared with traditional feedback [30].

Arthroscopy with biologic augmentation

Arthroscopic repair enables intra-articular pathology to be treated in a minimally invasive manner with biologic augmentation aimed at healing. Platelet-rich plasma (PRP) can provide concentrated growth factors, and bone marrow aspirate concentrate (BMAC) can provide mesenchymal stem/progenitor cells [29]. Scaffold-based implants provide a structural framework for tissue regeneration. PRP use intraoperatively during adolescent ACL reconstruction has been linked to faster graft ligamentization on MRI and less tibial tunnel widening at one-year follow-up (n = 92, prospective comparative study) [41]. Collagen-based scaffolds with microfracture in OCD lesions have demonstrated superior defect fill rates and patient-reported outcome measures at two years compared with microfracture alone [13]. The use of wearable technology and biologic augmentation is constrained in most community-based youth sports programs by the expense of the equipment, the inability to find trained data analysts, and the limited availability of biologics beyond special facilities [7]. Table 3 demonstrates new biological and technological interventions in the field of pediatric arthroscopic repair, identifying their possible advantages, clinical uses, and existing obstacles to mass deployment.

**Table 3:** Biologic augmentation and technology integration in pediatric arthroscopic repair The table was created by the authors using data summarized from the literature cited within the table ([40,19,38]). This table is an original synthesis and is not a verbatim reproduction of any published table; no publisher permissions are required.

Intervention	Study Context	Key Findings	Clinical Relevance	References
Platelet-Rich Plasma (PRP)	Adolescent ACL reconstruction; prospective comparative study (n = 92)	Faster graft ligamentization on MRI and reduced tibial tunnel widening at 1-year follow-up	Suggests PRP may enhance graft maturation and joint stability in skeletally immature athletes	[40]
Bone Marrow Aspirate Concentrate (BMAC)	Pediatric cartilage and ligament repair	Supplies mesenchymal stem/progenitor cells to support regeneration	May improve healing in cartilage lesions and ligamentous repairs	[19]
Collagen-Based Scaffold + Microfracture	Osteochondritis dissecans (OCD) lesions in children/adolescents	Higher defect fill rates and better patient-reported outcomes at 2 years vs. microfracture alone	Offers a more durable cartilage repair option for large or recurrent OCD lesions	[19]
Technology Integration – Wearables	Youth sports injury monitoring programs	Barriers: high cost, need for trained analysts, limited access to biologics in non-specialist centers	Limits adoption of advanced tech and biologics in community settings; underscores the need for accessibility strategies.	[38]

Regenerative medicine

Regenerative orthopedics involves the use of biological processes to replace the architecture and functions of musculoskeletal tissue. Within pediatrics, there is an emerging application in cartilage defects, non-union fractures, and some tendon or ligament injuries, but strong, long-term data are lacking.

Mesenchymal stem cells (MSCs) isolated in bone marrow, adipose tissue, or even in the umbilical cord have their therapeutic effect mainly through paracrine signaling by releasing growth factors and cytokines that regulate inflammation, attract host progenitor cells and induce angiogenesis [45]. PRP introduces a high level of platelet-derived growth factors to boost repair. Scaffolds typically are biodegradable matrices made of collagen or polymers and provide a 3D structure on which cells can attach and deposit an extracellular matrix [12]. Phase I/II study (n = 18 adolescents, follow-up 24 months): implantation of autologous bone marrow-derived MSCs in osteochondral lesions showed 83% complete defect filling at MRI, increased International Knee Documentation Committee (IKDC) scores, and no severe adverse events [46]. Pediatric microfracture augmented with collagen scaffold has also demonstrated an increased amount of hyaline-like repair tissue compared to fibrocartilage that occurred with microfracture alone in matched cohort studies [33].

Although promising results are reported in the short term, it has not been established whether the biologic repairs in growing joints last. Registries in adults indicate that scaffold-augmented cartilage repairs are functional more than 10 years after repair; there are no similar long-term data in children [47]. Also, MSC treatment is still costly, and regulatory provisions regarding cell processing differ significantly across jurisdictions, preventing multicenter pediatric trials [48]. The technological arc here, precision diagnostics in CP (gait analysis), advanced imaging in oncology (PET/MRI), biomechanical monitoring in sports (IMUs), and biologic augmentation in tissue repair, is an indication of the same principle: interventions are becoming more data-driven, less invasive, and more biologically integrated [49]. The examples of surgical practice, such as single-event multi-level surgery, 3D-printed expandable endoprostheses, biologically augmented arthroscopy, and scaffold-based cell therapies, demonstrate the combination of engineering, materials science, and molecular medicine in pediatric orthopedics [50]. The difficulty in all areas is providing equitable access to the world, standardizing pediatric-specific protocols, and creating long-term outcome evidence to support the sophistication of these innovations.

Limitations and future recommendations

Although there has been significant progress in the areas of diagnostic imaging, surgical innovation, and biologically directed therapies, there are a number of limitations that restrict the widespread use of such interventions in pediatric orthopedics. Most high-precision modalities, including low-dose CBCT and 3DST, are limited to the tertiary centers in high-resource settings due to the specialized equipment and operator experience that each entails [51]. The major drawbacks to PET/MRI use are the high capital expenditure and low availability of scanners, whereas magnetically controlled growing rods are limited by the cost of the device and specialized surgical training. There are other obstacles to cell-based regenerative therapies, such as regulatory demands, cell processing capacity variability, and manufacturing costs. Systematic reviews show that there is a paucity of pediatric-specific RCTs, and much of the clinical decision-making is based on extrapolation of adult evidence. In addition, meta-analyses of surgical and biologic interventions have median follow-ups of less than five years, which constrain insight into durability, growth-related outcomes, and late complications. Variation in surgical ability and local institutional practice also hinders reproducibility and inter-regional comparison of results.

In the future, more multicentric, prospective pediatric RCTs with standardized outcome measures, which involve functional recovery, quality of life, and cost-effectiveness, should be prioritized. To assess the long-term safety and performance of new interventions, longitudinal registries of real-world data are required that cover childhood, skeletal maturity, and adulthood. Implementation science needs to be used to develop flexible models of care to incorporate these technologies into various healthcare systems with specific capacity-building programs in low- and middle-income countries. Simultaneously, fundamental research such as biomechanical testing of implants in growing bone, studies of biomaterials unique to pediatrics, and molecular studies of how growth plates interact with tissue will help to design safer and more effective implants. A global, coordinated research agenda linking clinical innovation to equitable access will be essential to transforming these technological advantages into long-term improvements in pediatric orthopedic outcomes.

## Conclusions

Pediatric orthopedic care demands an integrated approach that unites diagnostic precision, biologic innovation, and surgical advancement to preserve growth potential and functional integrity. This review underscores the cross-disciplinary convergence of technologies such as AI-assisted imaging, regenerative biologics, and patient-specific implants, highlighting their collective potential to transform musculoskeletal care in children. However, sustained progress depends on robust, pediatric-specific evidence generation through multicentric trials and long-term registries that validate safety, efficacy, and developmental outcomes. By aligning innovation with accessibility and implementation equity, this review outlines a global roadmap for translating technological sophistication into practical, inclusive, and sustainable pediatric orthopedic care.
